# Laparoscopic Repair of a Ureteric Sciatic Hernia: Report of a Case

**DOI:** 10.1155/2014/787528

**Published:** 2014-02-23

**Authors:** Yasuo Tsuzaka, Kazuhiro Saisu, Nobuo Tsuru, Yukio Homma, Hiroyuki Ihara

**Affiliations:** ^1^Department of Urology, Shintoshi Hospital, 703 Nakaizumi Gotten, Iwata, Shizuoka 438-0078, Japan; ^2^Department of Urology, Juzen Hospital, 1975 Hiraguchi, Hamakita-ku, Hamamatsu, Shizuoka 434-0041, Japan; ^3^Department of Urology, The University of Tokyo Hospital, 7-3-1 Hongo, Bunkyo-ku, Tokyo 113-8655, Japan

## Abstract

Ureteric sciatic hernias are extremely rare. Here we report a case of a 78-year-old woman presented with colicky left abdominal pain. Computed tomography revealed a ureteric sciatic hernia, and drip infusion pyelography revealed dilated left ureter with herniation of the ureter into the sciatic foramen. The hernia was successfully repaired laparoscopically. We have described the diagnosis and management of the patient, followed by a review of the literature on sciatic hernias.

## 1. Introduction

In general, sciatic hernias are very rare with very limited literature worldwide [1]. Of all sciatic hernias, ureteric sciatic hernia is extremely rare. To the best of our knowledge, there have been only approximately 25 cases of ureteric sciatic hernias previously reported [1–5]. Successful laparoscopic management of ureteric sciatic hernia has been described for only three patients [2–4]. Here we report the fourth case of a laparoscopically repaired ureteric sciatic hernia.

## 2. Case Report

**Figure 1:**
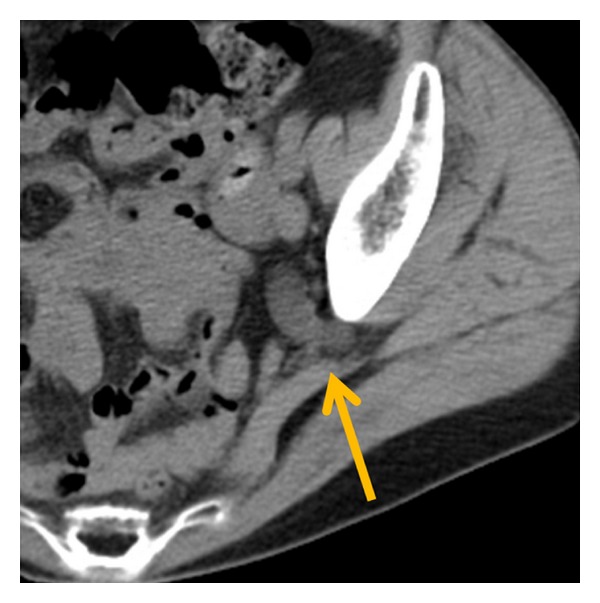
Computed tomography showed ureteral herniation into the sciatic foramen (arrow).

**Figure 2:**
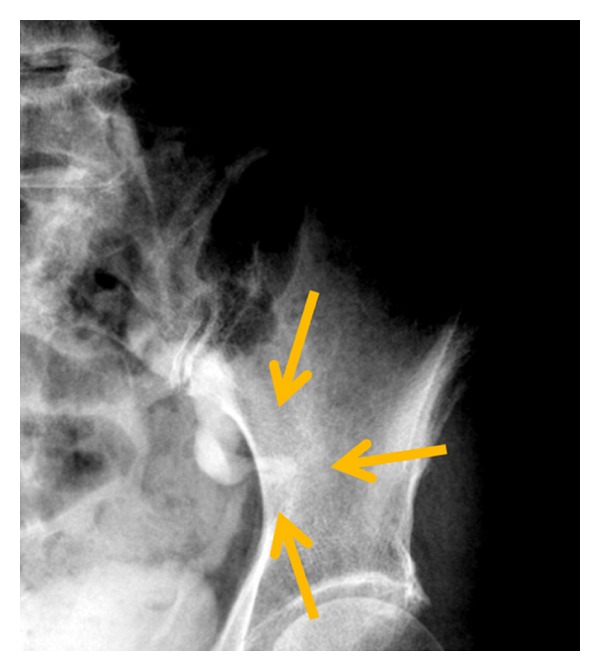
Drip infusion pyelography revealed “curlicue ureter” sign as the knuckle of the herniated ureter passed laterally to the medial wall of the bony pelvis (arrows).

A 78-year-old Japanese woman presented to our hospital because of colicky left abdominal pain. She had no history of any hip or neuromuscular disease. Her past medical history was only a Cesarean section. Physical examination revealed her height as 155 cm and weight as 35 kg (body mass index 14.5), and she was afebrile and in good general health. Blood examination and urinalysis was normal. She complained of left costovertebral angle tenderness. Computed tomography (CT) revealed dilated left ureter with herniation of the ureter into the sciatic foramen (Figure 1). Drip infusion pyelography (DIP) identified that the left ureter was dilated and ran an unusual and convoluted course laterally through the pelvis (Figure 2). We diagnosed her case as left ureteric sciatic hernia on the basis of these findings.

The patient's symptoms spontaneously are resolved before long. We followed up her closely without treatment for four months. However, the patient's symptoms recurred occasionally, and the left hydroureter remained unchanged. We decided to laparoscopically repair the hernia.

*Operative Management. *The patient was positioned in a head-down right semilateral position after general anesthesia. A balloon trocar was placed superior to the umbilicus. Three accessory ports (5 mm) were placed under direct laparoscopic control, and a three-dimensional (3D) laparoscopy system (Shinko Optical, Tokyo, Japan) was used. The peritoneum overlying the common iliac artery was elevated and transected, and the left ureter was identified on the artery. The ureter was mobilized all the way to the sciatic foramen by blunt dissection. The herniated ureter was reduced with light traction. A small defect was identified and repaired by suturing the edges of the surrounding connective tissue in order to close the hernia sac. The patient had an uneventful recovery. A month after the laparoscopic repair, DIP revealed that the left ureter had returned to its normal anatomical position without ureteral obstruction. Eight months after the surgery, the patient remains asymptomatic.

## 3. Discussion

Sciatic hernias are very rare, and a limited number of reports have been published worldwide. Sciatic hernia contains the ovary, ureter, small intestine, colon, neoplasm, greater omentum, and urinary bladder. Of all sciatic hernias, ureteric sciatic hernia is extremely rare. To the best of our knowledge, studies have previously reported only approximately 25 cases of ureteric sciatic hernias [1].

In ureteral herniation, ureter prolapses occur through the sciatic notch, which is divided by the sacrospinous ligament into the greater and lesser sciatic foramina. The greater sciatic foramen is subdivided by the piriform muscle into the suprapiriformis and infrapiriformis foramina, and it is considered to be a potential space because the piriformis muscle completely occupies the greater sciatic foramen [2]. Reports have suggested that atrophy of the piriformis muscle, which is caused by neuromuscular or hip joint diseases, is the predisposing factor for the development of ureteric sciatic hernia, creating a potential space through which the ureter migrates [2]. Sciatic hernias seem to occur more frequently in females because of their larger pelvis and sciatic foramina [6]. In our case, the patient had not previously suffered from any hip or neuromuscular diseases, and her body mass index was 14.5. A decrease in muscle mass may be involved in the development of ureteric sciatic hernia.

The clinical presentation of sciatic hernias is variable, and it can be acute or chronic. Symptoms range from none to life-threatening strangulation, and they reflect the size, location, and contents of the hernia [1]. Patients with ureteric sciatic hernias may experience symptoms of renal colic due to ureteral obstruction. Several cases have reported that ureteral sciatic hernias cause pyelonephritis and severe urinary sepsis [3].

CT is helpful in diagnosing sciatic hernias. Furthermore, excretory urography is also useful in diagnosing ureteric sciatic hernias. A curling ureter, also referred to as a “curlicue ureter” sign, is considered pathognomonic. The condition is best observed in the frontal projection on intravenous pyelography, wherein the knuckle of the herniated ureter passes laterally to the medial wall of the bony pelvis. Ureteric sciatic hernias may occasionally escape detection because the herniation may be intermittent [1].

In most cases, open surgical correction is performed, including a manual reduction of the hernia, and in some cases, grossly deformed ureters requires resection with reimplantation. The hernial defect has been previously handled using multiple methods. Closure of the defect by ligation and suturing of the sac or patching across the prosthetic mesh is performed to avoid recurrence of the hernia. In some cases, no repair is performed in asymptomatic patients [2]. No literature exists that provides reliable long-term dates to compare the results of one type of repair with another.

In 1999, Gee et al. first demonstrated successful laparoscopic management of ureteric sciatic hernia [2]. To the best of our knowledge, this is the fourth report of laparoscopically repairing a ureteric sciatic hernia. Although the laparoscopic repair was relatively straightforward in our patient, surgical exposure may be more challenging in other patients. However, with the relatively small incisions at four port sites, these patients receive the benefit of a faster recovery with less pain and better cosmetic result. We consider that laparoscopic surgical repair of a ureteric sciatic hernia is an effective alternative to open surgery.

Because ureteric sciatic hernias are extremely rare, no literature has provided a reliable long-term prognosis. In our case, laparoscopic repair was successfully performed, and there has been no evidence of a recurrent hernia in eight months postoperatively. Further case reports for ureteric sciatic hernias are required to determine the optimum management and treatment.
